# Pulse wave-based evaluation of the blood-supply capability of patients with heart failure via machine learning

**DOI:** 10.1186/s12938-024-01201-7

**Published:** 2024-01-19

**Authors:** Sirui Wang, Ryohei Ono, Dandan Wu, Kaoruko Aoki, Hirotoshi Kato, Togo Iwahana, Sho Okada, Yoshio Kobayashi, Hao Liu

**Affiliations:** 1 Graduate School of Science and Engineering, Chiba University, Chiba, Japan; 2 Department of Cardiovascular Medicine, Chiba University Graduate School of Medicine, Chiba, Japan

**Keywords:** Pulse wave, Blood-supply capability, Heart failure, Machine learning

## Abstract

Pulse wave, as a message carrier in the cardiovascular system (CVS), enables inferring CVS conditions while diagnosing cardiovascular diseases (CVDs). Heart failure (HF) is a major CVD, typically requiring expensive and time-consuming treatments for health monitoring and disease deterioration; it would be an effective and patient-friendly tool to facilitate rapid and precise non-invasive evaluation of the heart’s blood-supply capability by means of powerful feature-abstraction capability of machine learning (ML) based on pulse wave, which remains untouched yet. Here we present an ML-based methodology, which is verified to accurately evaluate the blood-supply capability of patients with HF based on clinical data of 237 patients, enabling fast prediction of five representative cardiovascular function parameters comprising left ventricular ejection fraction (LVEF), left ventricular end-diastolic diameter (LVDd), left ventricular end-systolic diameter (LVDs), left atrial dimension (LAD), and peripheral oxygen saturation (SpO_2_). Two ML networks were employed and optimized based on high-quality pulse wave datasets, and they were validated consistently through statistical analysis based on the summary independent-samples *t*-test (*p* > 0.05), the Bland–Altman analysis with clinical measurements, and the error-function analysis. It is proven that evaluation of the SpO_2_, LAD, and LVDd performance can be achieved with the maximum error < 15%. While our findings thus demonstrate the potential of pulse wave-based, non-invasive evaluation of the blood-supply capability of patients with HF, they also set the stage for further refinements in health monitoring and deterioration prevention applications.

## Introduction

Heart failure (HF) has become a significant health concern affecting approximately 26 million people worldwide, particularly older adults who normally require lifelong treatment [1–3]. HF is characterized as “a condition in which the heart cannot adequately pump blood to fulfill the body’s requirements” or “a condition leading to an abnormality in cardiac structure or function that results in the failure of effective oxygen transport for metabolic requirements” [1]. HF is clinically diagnosed using the Framingham criteria, which are primarily used in most research [4]. Because patients with HF have poor blood circulation throughout their bodies, most of them suffer from concurrent cardiovascular diseases from an early stage [5]. The cardiac chambers of patients with HF are generally morphologically remodeled, causing dysfunctions with a significant decline in the pump function of the heart and reduced blood-supply capability [6–9]. Consequently, the oxygen levels in the arterial blood vessels throughout the body decrease, showing symptoms, such as shortness of breath, fatigue, weakness, and decreased exercise capability, which severely affect the patients’ daily lives and necessitate long-term medication to maintain normal daily activities [10].

Patients with severe HF require hospital visits for prompt medical diagnosis and comprehensive evaluation by clinicians. Various physiological signals and medical images are normally obtained using medical devices, such as the echocardiography test, which is the most standard tool to assist physicians in assessing patients’ conditions every three to six months [11]. Digital imaging of cardiac chambers is crucial for evaluating the blood-supply capability in patients with HF, including the left ventricular ejection fraction (LVEF), left ventricular end-diastolic diameter (LVDd), left ventricular end-systolic diameter (LVDs), and left atrial dimension (LAD), because patients with HF show irregular characteristics of lower LVEF and higher values of LVDd, LVDs, and LAD [5, 12]. However, echocardiography tests are time consuming and expensive, posing challenges for patients with HF [13]. For instance, in the United States, even a 45 min to 1 h echocardiography test may cost approximately 2000 dollars for a patient, and it is unavailable for the daily monitoring of patients with HF [14–16]. When patients temporarily suffer from severe chest pain, fainting, weakness, arrhythmia, or severe shortness of breath [17], a timely diagnosis of their blood-supply capability to appropriately decide on a medical intervention is crucial to avoid overtreatment and prevent deterioration. Thus, it is necessary to utilize the limited medical resources for accomplishing real-time home health monitoring of patients with HF and providing them with a timely deterioration warning.

Physiological signals, such as pulse waves, have been widely used for health monitoring and disease prediction [18–23]. Pulse waves provide vital physiological information associated with the blood-supply capability and delivery efficiency [24, 25]. The non-invasive and convenient nature of pulse wave measurements allows the employment of various low-cost home electronic devices for the initial diagnosis of cardiovascular diseases and related complications [26–28]. Considering that the abnormal heart chamber geometry typically observed in patients with HF alters the ejection fraction, ultimately impacting blood production and delivery efficiency [5], it would be an effective and patient-friendly tool to achieve non-invasive assessments of the heart's blood-supply capability through physiological and pathological information embedded in pulse waves. Such assessments provide significant potential for health monitoring and prevent disease deterioration in patients with HF.

**Fig. 1:**
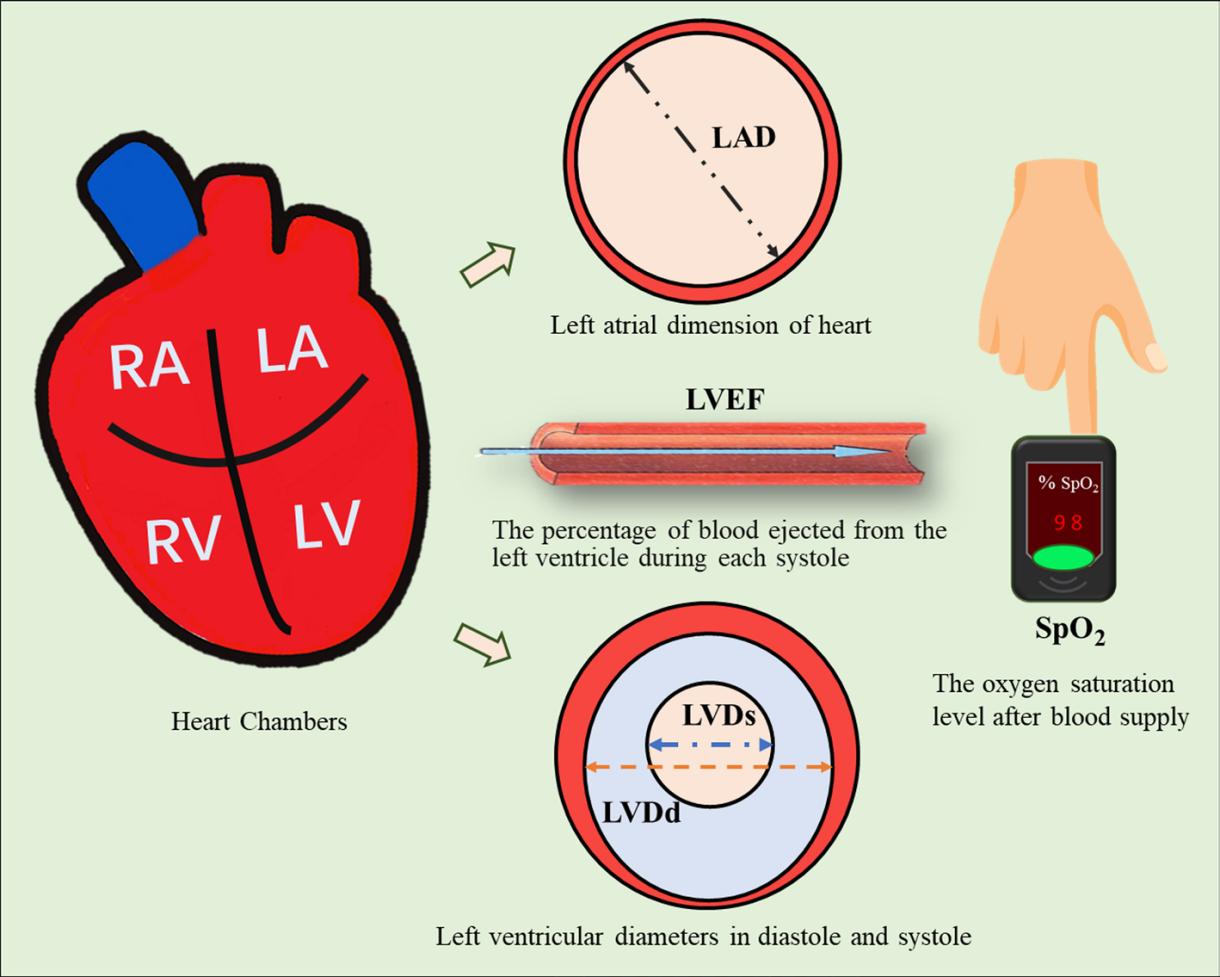
Schematic of blood-supply capability. *RA* right atrium, *LA* left atrium, *RV* right ventricle, *LV* left ventricle, *SpO*_*2*_ peripheral oxygen saturation, *LVDd* left ventricular end-diastolic diameter, *LVDs* left ventricular end-systolic diameter, *LVEF* left ventricular ejection fraction, *LAD* left atrial dimension

Although quantitative analysis of pulse wave signals has been applied to certain cardiac functions or specific diseases [13, 18, 29], previous studies only targeted healthy subjects and other patients without HF. In particular, the quantitative evaluation of the pulse wave-based blood-supply capability of patients with HF remains unexplored [30]. Owing to the uncertainties caused by the noise and interference generated in the pulse-wave sampling process [31], such pulse wave-based prediction of blood-supply capability is normally restrained by the limitations of conventional qualitative statistical methods [32, 33]. To establish a fast and non-invasive strategy for effectively predicting the blood-supply capability of patients with HF, we proposed a machine learning (ML)-based model in this study to predict five representative cardiovascular function parameters associated with the heart’s blood-supply capability [34]. As illustrated in Fig. 1, the parameters, i.e., LVDd, LVDs, and LAD, directly evaluate the morphological condition of the heart chamber and the heartbeat functions at systole and diastole; the LVEF quantifies the ratio of blood supply from the heart; and the SpO_2_ determines the patient’s blood oxygen level at the end of the blood supply as well as the supply efficiency. It has been broadly recognized that the ML methodology has powerful and feasible capabilities in robust feature extraction [33, 35–39]. Remarkable achievements have been accomplished in various research fields, such as intelligent medicine, medical image processing, and autonomous driving, by integrating multiple basic features into complex features, enabling the mapping of the image or multi-dimensional signal data onto different prediction targets [40–44]. Our previous study verified that the ML-based strategy enabled the fast and efficient prediction of cardiac functions based on peripheral pulse waves [45], demonstrating the high potential and capability of multilayer feature extraction in accurately predicting the relevant indicators for clinical application owing to ML methods. In this study, we further explored the capability and feasibility of ML-driven, pulse wave-based prediction of the blood-supply capability of patients with HF for clinical application.

## Results

After critical screening and preprocessing of the pulse wave data of the 237 patients with HF, a high-quality ML dataset was successfully constructed in a suitable manner for flexible input data formats and datasets. To ensure that the screened patients fit in the clinical statistics of the patients with HF associated with the five parameters (SpO_2_, LVDd, LVDs, LVEF, and LAD) for evaluating blood-supply capability and the relevant clinical information summarized in Table 1, we conducted a summary independent-samples *t*-test based on the statistical results and a comparison with reliable data of previous studies [46–48]. Our results showed good consistency in terms of *p* value (*p* > 0.05) (Table 1) in the test analysis for the five parameters and other physiological information.

**Table 1:** Characteristics of patients with heart failure (HF)

Characteristics	Range	Others’ reports	*p* values
Age (years old)	66.4 ± 16.4	68.0 ± 15.0	0.162
BMI	24.2 ± 5.7	23.5 ± 3.9	0.076
BPs	129 ± 35.4	133.0 ± 29.9	0.105
BPd	80.2 ± 22.7	79.6 ± 18.6	0.703
HR	76.8 ± 15.1	76.0 ± 14.0	0.447
SpO_2_	96.9 ± 2.6	97.2 ± 1.8	0.501
LVDd	56.8 ± 10.7	55.9 ± 14.9	0.604
LVDs	46.5 ± 12.9	48.7 ± 11.9	0.153
LVEF	38.3 ± 15.3	38.0 ± 15.0	0.779
LAD	44.4 ± 9.1	42.3 ± 9.4	0.074

Data are presented as mean ± SD

*BMI* blood mass index (kg/m^2^), *BPs* blood pressure systole (mmHg), *BPd* blood pressure diastole (mmHg), *HR* heart rate (beats/min), *SpO*_*2*_ peripheral oxygen saturation (%), *LVDd* left ventricular end-diastolic diameter (mm), *LVDs* left ventricular end-systolic diameter (mm), *LVEF* left ventricular ejection fraction (%), *LAD* left atrial dimension (mm)

**Fig. 2:**
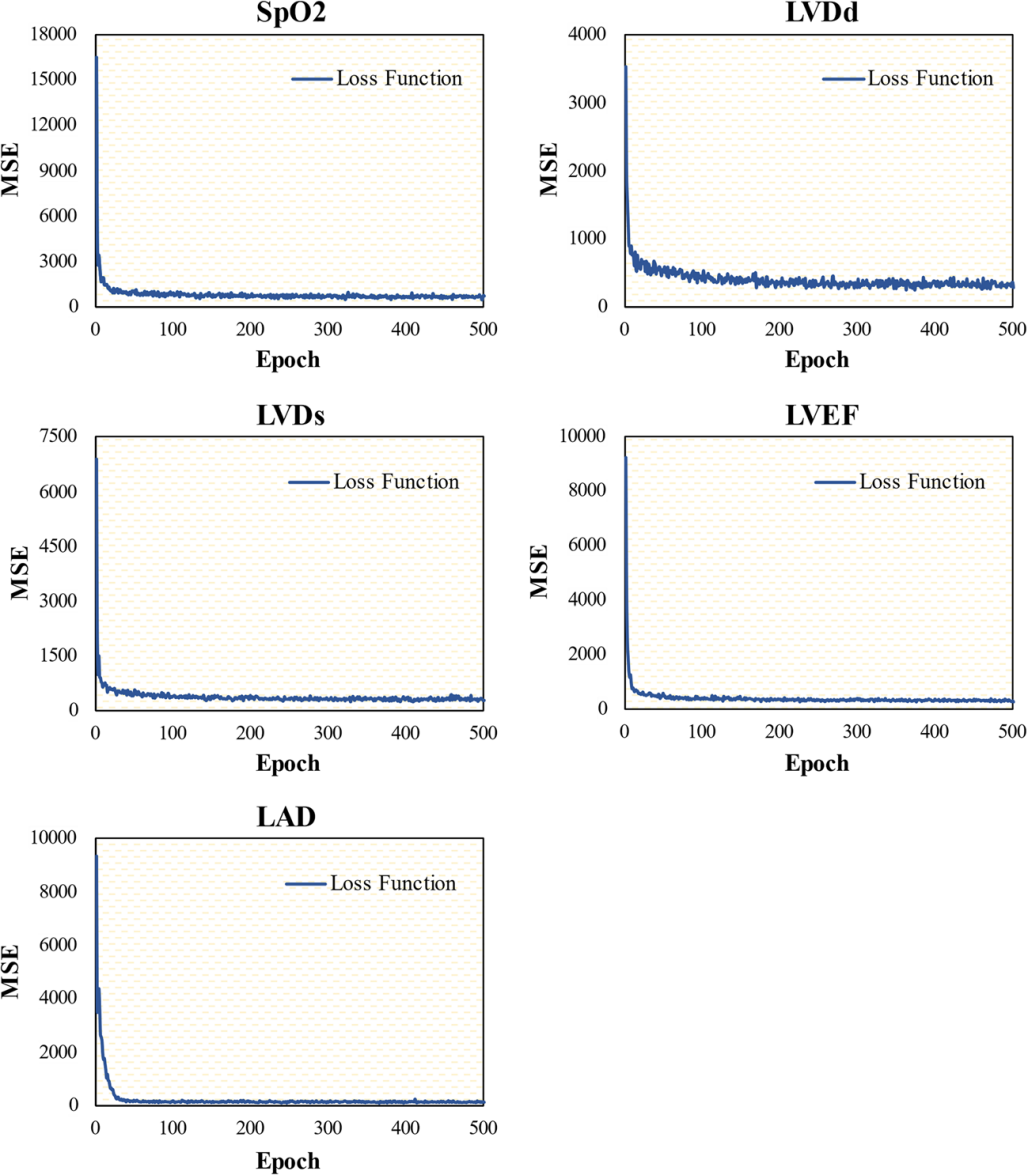
Illustration of DenseNet model-based learning curves from the training set for blood-supply capability parameters of peripheral oxygen saturation (SpO_2_), left ventricular end-diastolic diameter (LVDd), left ventricular end-systolic diameter (LVDs), left ventricular ejection fraction (LVEF), and left atrial dimension (LAD)

Five parameters were used to evaluate the blood-supply capability of the two selected ML structure models. During ML training, 10 network optimizations were accomplished, in which reduction in the loss function MSE resulted in a rapid and monotonous decline in each epoch. Using DenseNet as an example, as shown in Fig. 2, the MSE curves for every evaluation parameter with a training epoch of 500 exhibited a constantly decreasing trend to the minimum level. This indicates that the relevant parameters and weights of the network were eventually optimized when the training process converged to a stable stage, which was then stored for ML-based testing. Moreover, the MSE curves of the fully connected network exhibited a decreasing trend, similar to that of DenseNet. In the test phase, both ML models outputted the predicted values for the five parameters within 1 s using the input of the pulse wave signals in the test set.

To examine the predictive performance of the two networks on the five parameters, we summarized the error function results in Table 2. The DenseNet model consistently shows lower MAPE values, indicating more accurate predictions. For SpO_2_, DenseNet and the fully connected network achieve MAPEs of 5.6% and 6.6%, respectively. In measuring left ventricular dimensions, DenseNet reports MAPEs of 12.9% for LVDd and 14.6% for LVDs, compared to 14.7% and 17.3% by the fully connected network. A significant difference is observed in the prediction of LVEF, where DenseNet has a MAPE of 18.2%, lower than the fully connected network's 21.2%. For LAD, the MAPEs are 12.0% for DenseNet and 14.9% for the fully connected network, further indicating DenseNet's enhanced predictive capability for blood-supply indicators.

**Table 2:** Comparison between the predictions of the two machine learning models for the values of the five parameters associated with blood-supply capability

ML networks	Error function	Predicted values				
		SpO_2_ (%)	LVDd (%)	LVDs (%)	LVEF (%)	LAD (%)
Fully connected network	MAPE	6.6	14.7	17.3	21.2	14.9
DenseNet		5.6	12.9	14.6	18.2	12.0

*SpO*_*2*_ peripheral oxygen saturation, *LVDd* left ventricular end-diastolic diameter, *LVDs* left ventricular end-systolic diameter, *LVEF* left ventricular ejection fraction, *LAD* left atrial dimension, *MAPE* mean absolute percentage error

**Fig. 3:**
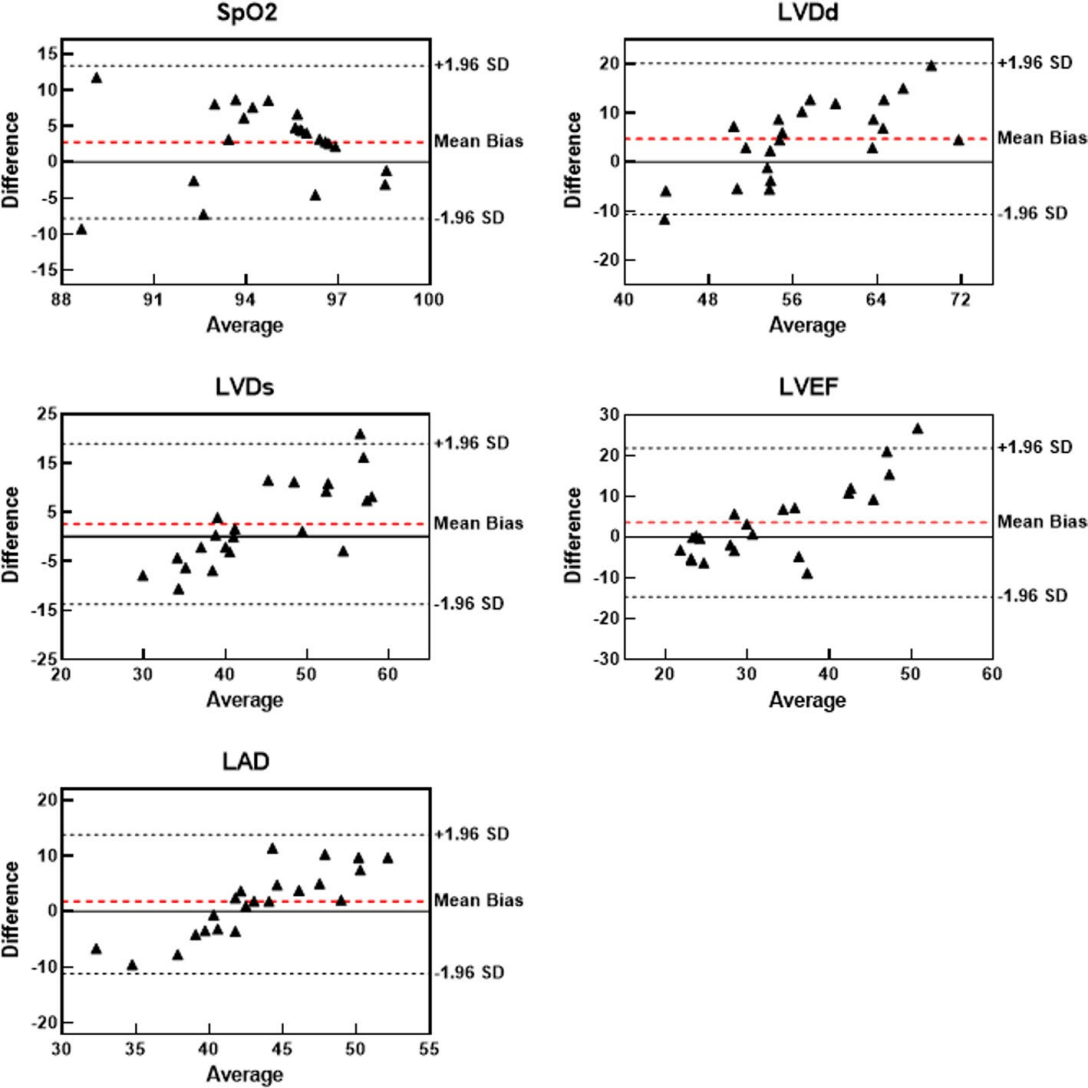
Bland–Altman analyses between DenseNet-based predictions and clinical measurements for five parameters in terms of peripheral oxygen saturation (SpO_2_), left ventricular end-diastolic diameter (LVDd), left ventricular end-systolic diameter (LVDs), left ventricular ejection fraction (LVEF), and left atrial dimension (LAD)

**Fig. 4:**
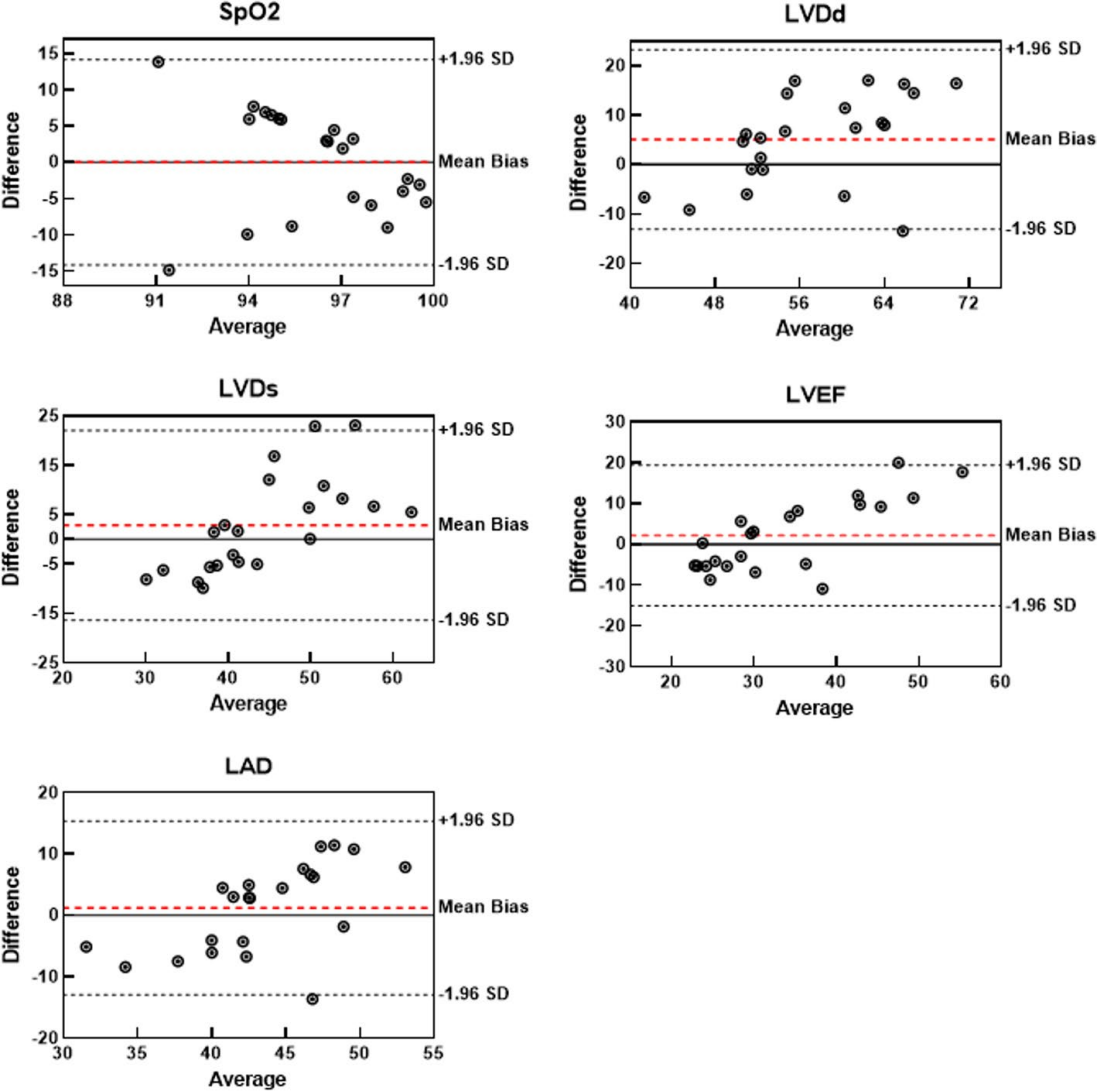
Bland–Altman analyses between fully connected network-based predictions and clinical measurements for five parameters in terms of peripheral oxygen saturation (SpO_2_), left ventricular end-diastolic diameter (LVDd), left ventricular end-systolic diameter (LVDs), left ventricular ejection fraction (LVEF), and left atrial dimension (LAD)

We further applied the Bland–Altman method to assess the consistency between the ML predictions and clinical measurements by analyzing the average values and mean bias, which were visualized in a scatter plot, as shown in Figs. 3 and 4, where the horizontal and vertical axes represent the average value and the difference [with a 95% distribution range, that is, the confidence interval (CI)], respectively. Good consistency between the two methods occurs when the points within a CI of the scatter plot account encompassing over 95% of all points, with the CI not exceeding the range of critical values for clinical applications [49]. Most sets of the predictions of the two ML networks were within the 95% CI. For the DenseNet, those excluding the sets of LAD were fell into the 95% CI, other parameters (i.e., SpO_2_, LVDs, LVEF and LVDd) had only one set of data samples outside the 95% CI. For the fully connected network, although the LVDs had two sets not within the 95% CI, the other parameters (SpO_2_, LVDs, LAD, and LVDd) contained only one set outside the 95% CI. Thus, the ML network-based predicted results agreed well with the clinical measurements.

## Discussion

In this study, we successfully applied ML method to perform a non-invasive evaluation of blood-supply capability through pulse wave signals without performing echocardiography in patients with HF. Our model takes an averaged pulse waveform as the input and predicts specific scalar values for parameters like LVEF, LVDs, LVDd, LAD, and SpO_2_ and has verified that ML networks have high potential and feasibility for achieving good performance in predicting five cardiovascular function parameters. In clinical practice, patients with HF have various cardiac functions that distinguish them from healthy people, making it difficult for expert physicians to make a reasonable diagnosis. Therefore, the ML model-based evaluation methodology developed in this study can be used as a fast and effective tool to assist physicians in providing patient-specific diagnoses and medical treatments. Moreover, LVEF and LAD are crucial factors for physicians to determine the indication for treatment; for instance, patients (less than 40%) with LVEF are normally recommended to use cardioprotective medications, whereas LAD is important for ablation therapy in patients with HF having atrial fibrillation [1].

The dataset used in this study was collected only from patients with HF without information on healthy subjects, and physiological information such as age and other cardiovascular function-related parameters may interfere with pulse wave signals after data screening, as pointed out by Scolletta et al. in a study of the correlation between pulse waves and LVEF [50]. To resolve the issue on methodological consistency associated with the ML model-based analysis, we performed a summary independent-samples *t*-test to validate the filtered data-based results with reliable datasets. The DenseNet model achieved a high-prediction performance owing to rigorous data screening, which was verified to be capable of successfully ruling out the potential interference of numerical discrepancies in the datasets used for ML analysis, thus ensuring the validity and quality of the datasets.

This exploration is particularly salient in the context of heart failure patients undergoing routine health monitoring. The ML model developed here not only identifies quantifiable relationships between easily measurable signals and critical health parameters but also holds the potential to provide early alerts to medical practitioners, streamlining timely interventions. As these patients often lack access to advanced diagnostic tools, such proactive diagnostic capabilities can bridge the crucial diagnostic gap and facilitate timely patient-specific treatments. Furthermore, while there are existing studies utilizing ECG signals for prediction of related parameters via ML method [51, 52], our approach leverages pulse wave signals, which are notably less expensive and easier to obtain, thus potentially revolutionizing the daily monitoring and deterioration prevention in heart failure patients. Clinical measurements of blood-supply capability parameters are normally performed using various expensive and high-tech medical devices under the guidance and operation of highly skilled technicians, which likely hinders patients with HF from receiving timely diagnosis and monitoring. Although configurations of ML networks, such as epochs, batch size, and the Adam optimizer, require a considerable amount of training and testing, as well as manual adjustments to improve the prediction accuracy and performance, the ML-based strategy proposed here can reduce the time, cost, and usage of medical devices. In addition, recent portable and multifunctional electronic devices such as smartwatches and smartphones have been innovating non-invasive measurements of various physiological signals in a more convenient and cost-effective manner [53–56]. Therefore, using these portable electronic devices, the pulse wave-based ML methodology proposed here could provide an inexpensive and patient-friendly tool to achieve fast and accurate evaluation of the blood-supply capability of patients with HF for real-time monitoring and diagnosis.

The pulse wave-based ML strategy was verified to have high clinical potential and feasibility. Based on the pulse wave signals of 237 patients with HF, together with clinical information on their heart’s blood-supply capability, a high-quality dataset was constructed after rigorous data screening and preprocessing of the pulse waves. Using five selected cardiovascular function parameters: LVEF, LVDd, LVDs, LAD, and SpO_2_, which were based on pulse waves using the fully connected network model and DenseNet model, **t**he non-invasive predictions agreed well with the clinical measurements. The prediction performance, which was evaluated through statistical analysis in terms of the error function and consistency, indicated that the proposed ML model achieved a highly accurate prediction (MAPE < 13%) for LVDd, LAD, and SpO_2_. In achieving the optimal predictive performance with the limited dataset, we acknowledge that our hyperparameter tuning was constrained to grid and random searches without the utilization of a separate validation set. This constraint will be addressed in future studies by incorporating more rigorous validation processes, like k-fold cross-validation or expanding the dataset for a dedicated validation set, enhancing the robustness and generalizability of our model.

While our approach shows promise, its foundation on a dataset has certain limitations. The limitations of this study were mainly caused by the insufficiency of datasets in terms of the clinical parameter scope and data quantity in comparison with previous studies of HFs or physiological signal analyses [57–59]. Although we had a comparatively large and rigorously screened dataset, it was difficult to interpret whether the classification of patients with HF considering specific cardiovascular diseases, such as arrhythmia or heart valve problems, could improve or reduce the prediction performance of the ML models. In addition, other clinically important indicators, such as B-type natriuretic peptide, a hormone produced by the heart in response to increased pressure and volume that is commonly used for diagnosing patients with HF [60], have yet to be considered. In addition, the diversity of patients with HF may also be an essential factor affecting the generalizability and flexibility of our findings, because this study used patient data collected only from a single institution, and pulse wave signals were obtained from the same device. The demographic and clinical characteristics of the patient population [61] may alter the prediction performance of the ML methodology. We acknowledge the immense value of real-time prediction based on time series for clinical diagnostics. Acknowledging the limitations of our current dataset, our future work will focus on expanding the dataset to include diverse demographics and varied heart failure severity levels to enhance the model’s generalizability and robustness. While our current methodology diverges from this approach, we see potential synergies and avenues for integration in the future. To explore real-time health monitoring and deterioration prevention in patients with HF, our future task will focus on the optimization of the proposed ML networks, the use of larger datasets for training and testing, and incorporation of relevant clinical information. Additionally, we aim to expand our dataset to include a more diverse patient population, encompassing varied demographics and different levels of heart failure severity, to enhance the generalizability and robustness of our ML model. This will involve gathering data from multiple institutions and potentially from a multi-center study, to foster a model that can adapt and perform accurately across diverse patient groups. Moreover, understanding the dynamic interplay between these cardiovascular function parameters like LVEF, LVDd, LVDs, LAD, and SpO_2_ will be pivotal. By delving deeper into how these parameters influence each other in various scenarios, we can refine our model predictions and offer more comprehensive insights to clinicians. This multifaceted approach, taking into account not just the individual parameters but their intricate relationships, will undoubtedly pave the way for more precise and patient-specific diagnostic tools.

## Conclusions

We have proposed a machine learning (ML)-based strategy for pulse wave-based, non-invasive evaluation of the blood-supply capability in association with patients with heart failure (HL) compared with conventional echocardiography, which is verified capable of achieving fast and robust prediction of five cardiovascular function parameters of LVEF, LVDd, LVDs, LAD, and SpO_2_. The ML models developed based on either the optimized fully connected network and the DenseNet demonstrated significantly high performance through consistency and error analyses, and comparison with clinical measurements. Given that the five parameters, particularly the LVEF and LAD are crucial for physicians to make a medical treatment decision, the ML-based evaluating system is of high clinical potential and feasibility for health monitoring and deterioration prevention in patients with HF. Moreover, this methodology could be applied to various health monitoring applications, providing timely warnings and diagnosis for HF patients by means of various non-invasive, portable, and patient-friendly devices to record pulse wave signals. In the future, to enable real-time health monitoring and deterioration prevention for various patients with HF even suffering from specific cardiovascular diseases, such as arrhythmia or heart valve problems, we will make further improvement on the prediction performance by optimizing the ML networks while building up sufficiently large pulse wave datasets and accounting for more relevant clinical information as well.

## Materials and methods

### Clinical data acquisition and screening

All data used in this study were obtained from 237 patients with HF, including raw extremity pulse wave data and relevant clinical, physiological, and pathological information. HF was diagnosed based on the Framingham heart failure diagnostic criteria [4]. All participants were admitted to Chiba University Hospital between January 2019 and August 2021. After stabilizing the HF condition with treatments, patients were positioned supinely for measurements. The blood pressure and pulse wave data were recorded using the blood pressure/pulse wave detection equipment BP-203RPEIII (Omron Corporation, JP), with each session lasting approximately 5 min. Simultaneously, SpO_2_ was measured using a Nonin Onyx Vantage 9590 Finger Pulse Oximeter (Nonin Medical Inc., USA). All patients underwent transthoracic echocardiography (Vivid E9; GE Healthcare, Horten, Norway) within one week before or after the pulse wave tests, and experts of the echocardiography measured the values of LVEF, LVDd, LVDs, and LAD from the echocardiography test and relevant clinical information (e.g., age and body mass index (BMI)) was also collected. During hospitalization, none of the patients consumed spicy food or alcoholic drinks. While there is limited specific literature on the stability of echocardiographic parameters over such a short period, our patient cohort consisted of individuals with stabilized HF condition post-treatment, where significant variations in these parameters are less likely. However, we acknowledge this as a potential limitation that needs further investigation.

### Dataset creation

**Fig. 5:**
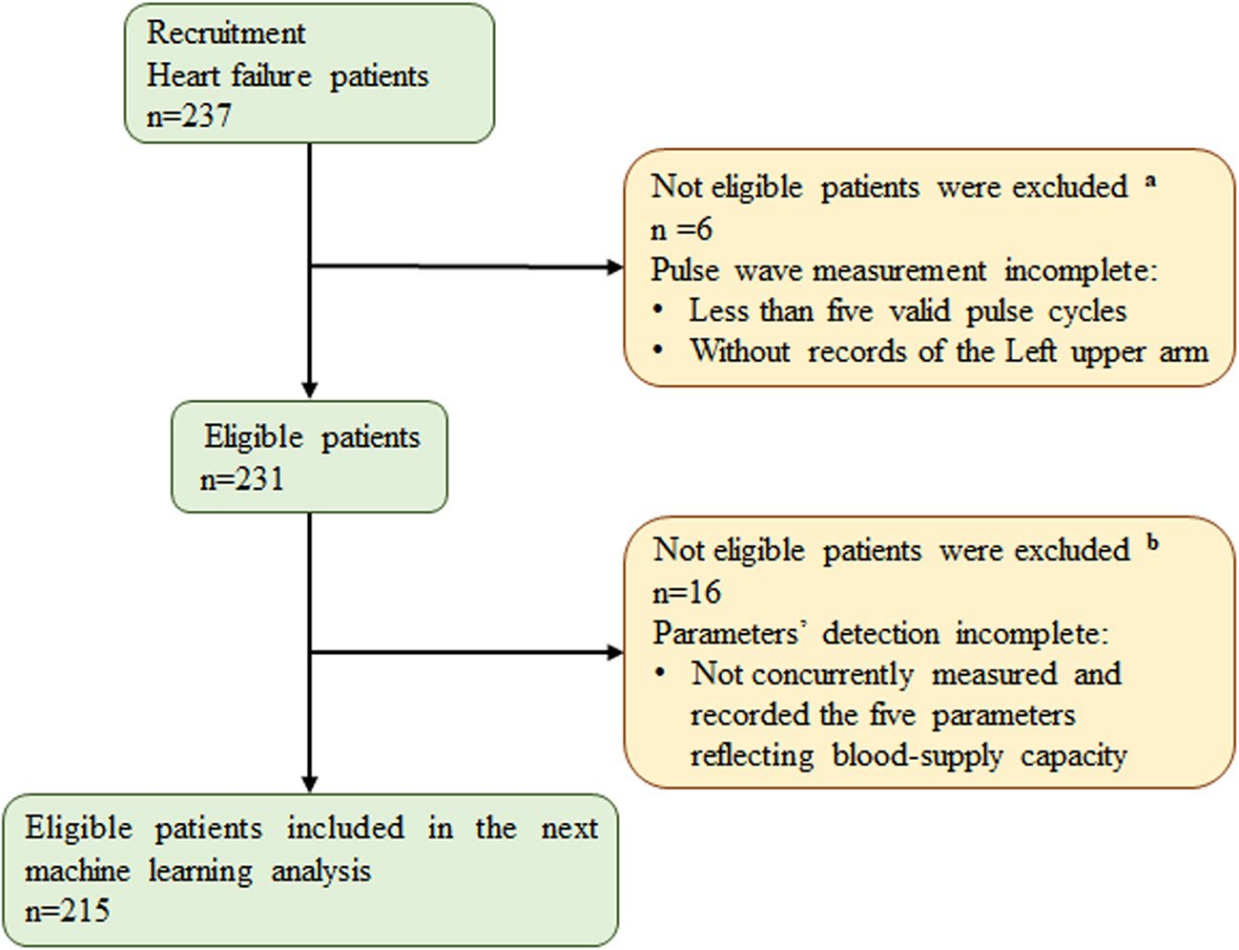
Flow-chart of patient screening. Screening criteria: (1) six patients without complete pulse wave measurement were excluded, and (2) 16 patients without simultaneous measurement and recording of the five parameters were excluded

As shown in Fig. 5, we performed rigorous data screening to ensure the quality of the data, and 215 patients with HF complied the following screening criteria: (1) the pulse wave data were collected from the left upper arm; (2) more than five valid pulse wave cycles were recorded; and (3) the five parameters (i.e., SpO_2_, LVEF, LVDd, LVDs, and LAD) associated with blood-supply capability were concurrently measured and recorded for each patient. To ensure the validity of the screened data, we applied the summary independent-samples *t*-test method to implement a statistical analysis of the consistency of the data with previous studies [46–48], which were examined in terms of the mean ± standard deviation, resulting in *p* > 0.05, thus a reasonable dataset [8, 62, 63].

The pulse wave data preprocessing methods developed in previous studies [64, 65] were employed to eliminate various noise and interference signals during the pulse wave sampling process: (1) using the averaged target pulse wave of over more than five valid heartbeat cycles in a steady state [66, 67], wavelet transform decomposition was conducted to remove noise [19, 45]; (2) as per Nyquist’s theorem, and considering a sampling frequency of 1000 Hz, the sampling nodes of pulse wave were converted from 1000 to 100; and 3) the pulse wave amplitude was normalized within a 0–100 scale. Subsequently, a pulse wave dataset was established from the data of 215 patients with HF, which was then segregated into training and testing datasets following a 9:1 ratio. The details of the dataset used for the ML analysis are encapsulated in Table 3.

**Table 3:** Machine learning datasets for five parameters

Parameters	SpO_2_	LVEF	LVDs	LVDd	LAD
Total number of included patients	215	215	215	215	215
Training set	193	193	193	193	193
Testing set	22	22	22	22	22

*SpO*_*2*_ peripheral oxygen saturation, *LVDd* left ventricular end-diastolic diameter, *LVDs* left ventricular end-systolic diameter, *LVEF* left ventricular ejection fraction, *LAD* left atrial dimension

### Machine learning network

**Fig. 6:**
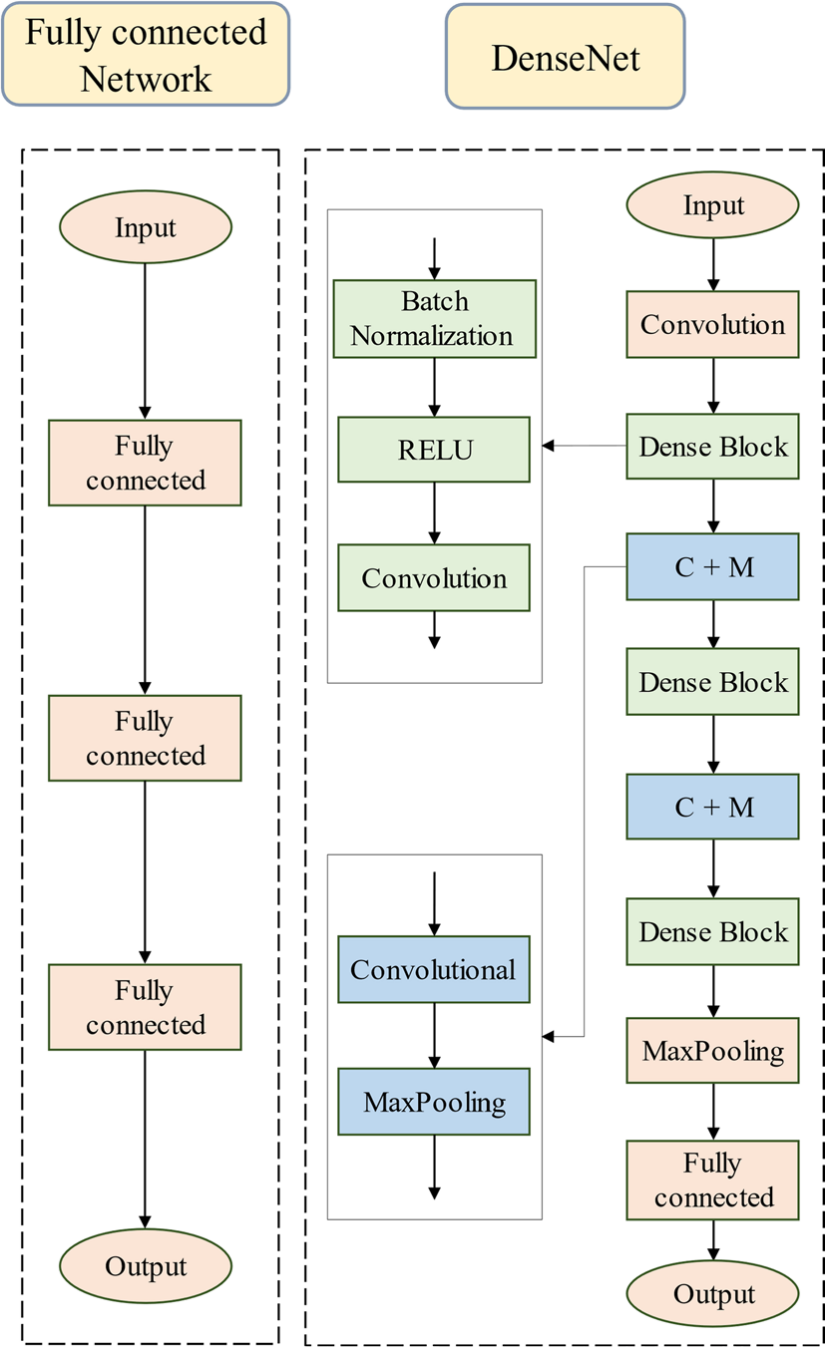
Structures of two machine learning networks

To clarify, each of the five parameters (i.e., SpO_2_, LVEF, LVDd, LVDs, and LAD) was trained separately using the ML networks. By predicting them independently, we aimed to ensure a focused and precise prediction for each clinical measurement without potential interference from the others. After conducting numerous preliminary tests to comprehensively compare the predictive performance among various ML networks (e.g., RNN and LSTM), we finally selected two optimized ML networks for further analyses, as shown in Fig. 6. It comprised a fully connected network and a DenseNet network [68, 69]. Fully connected networks are known for their efficiency and are a widely used tool in various investigative domains. On the other hand, DenseNet is a novel network that has been recently proposed and has exhibited outstanding performance in executing regression prediction tasks due to its effective feature extraction capabilities [70]. The selection was based on a critical evaluation of prediction performance, computational efficiency, and convergence speed, ensuring that the chosen networks were best suited for precise and efficient parameter prediction.

In the fully connected network, the input layer consisted of 100 neurons, which were identical to the sampling nodes of the input pulse waves. There were three fully connected layers with 256, 64, and 16 neurons. The output layer was composed of one neuron, with five evaluation parameters selected separately as the output of five training times. Except for the input layer, the calculation process for each neuron in the (*n* + 1)th layer of the fully connected network is described as:

1 $${{\text{Output}}}^{n+1 }=F\left(\sum_{j=1}^{{m}_{n}} {W}_{j}^{n+1}{{\text{Output}}}^{n}+{B}_{n}\right),$$

where *F* denotes the activation function ReLU, which was introduced to alleviate gradient vanishing during ML training and to accelerate the convergence of loss functions [71]. ${m}_{n}$ and ${B}_{n}$ are the number of neurons and the bias in the *n*th layer, respectively, and ${W}_{j}^{n+1}$ is the weight of the *j*th neuron in the *n*th layer.

The DenseNet that was verified to be capable of effectively increasing the usage of data features and achieving high performance even with limited data, while avoiding overfitting [70], shared the same input and output layers as in the fully connected network. The network contains three dense block modules to connect all the layers while transferring various features between the layers, and it begins with an initial convolution layer equipped with 64 kernels, each having a size of 3. This layer serves as the foundation for extracting primary features from the input data before it is passed through to the subsequent dense blocks. Following the dense blocks and transition layers, the network implements a MaxPooling layer with a pooling window of size 2 and a stride of 2 to reduce the spatial dimensions of the feature maps, which is aimed at reducing the computational complexity and helps in making the detection of features invariant to scale and orientation changes. After that, a fully connected layer consisting of 64 neurons, which is tasked with the role of regression, effectively mapping the extracted features to the output values. The input and output of the (*n* + 1)th layer (featured map) associated with the dense block module can be expressed as follows:

2 $${\text{Output}}^{n+1}=\text{ feature map }={G}^{n}\left({ \, {\text{Output}}}^{1},{ \, {\text{Output}}}^{2},\dots ,{ \, {\text{Output}}}^{n}\right),$$

where G denotes multiple operations including the ReLU, batch normalization, and convolution.

The training was carried out using the mean square error (MSE) as a loss function to evaluate the two ML networks combined with the Adam optimizer. The two ML networks were trained utilizing TensorFlow (v2.0.0rc, Python 3.7) on an NVIDIA GeForce GTX 1660 Ti GPU. Hyperparameter tuning was performed using a blend of grid and random searches to determine the optimal model settings, taking into account a trade-off between prediction performance and computational efficiency. During the ML training, the utilization of different amounts of data associated with the back-propagation algorithm for adjusting the parameter configuration of the network (e.g., the number of network layers and neurons) may lead to a decline in the loss function and an alteration in the prediction performance. As a result, the network structure size was adjusted to ensure promising convergence in the loss function during network training. Additionally, we employed the early stopping strategy, a common practice in ML to prevent overfitting during training process as well as the preliminary experiments, which we have previously detailed and visually represented in our published work [45, 72]. This strategy involves monitoring the predictive accuracy during training and ceasing further iterations when no improvement is observed for the predictive performance on the test dataset more than consecutive 50 epochs. The Adam optimizer was chosen under the following conditions: learning rate = 0.001, *ε* = 0.001, *ρ*1 = 0.9, *ρ*2 = 0.999, and *δ* = 1E−8 [73], and the epoch parameter was set to 500. After training, each loss function curve and the relevant optimal configuration of the networks associated with the five parameters (SpO_2_, LVEF, LVDd, LVDs, and LAD) were recorded and stored. For testing, the ML-predicted parameters were used for statistical analysis and comparison with clinical measurements.

### Performance evaluation

Following previous studies [45, 74, 75], we employed the mean absolute percentage error (MAPE) as the error function to verify the ML-based prediction for the test datasets:

3 $${\text{MAPE}}=\frac{\sum_{n=1}^{N} \left|\left({y}_{n}-{\widehat{y}}_{n}\right)\frac{1}{{y}_{n}}\right| \times 100\%}{N},$$

where ${y}_{n}$ and ${\widehat{y}}_{n}$ are the clinical-measured and ML-predicted values of the five parameters, respectively, and *n* is the size number of the test dataset.

In addition, the Bland–Altman method was used for consistency analysis of clinical measurements and ML-based predictions. The Bland–Altman can dissect the discrete trend, clustering tendency, and correlations of the five parameters between the two datasets of clinical measurements and ML-based predictions. When the five parameters fell within the permissible range, the two datasets were considered to possess good consistency, and the two methods could potentially replace each other [44, 76].

## Data Availability

Not applicable.

## Abbreviations

CVS: Cardiovascular system
CVDs: Cardiovascular diseases
HF: Heart failure
ML: Machine learning
LVEF: Left ventricular ejection fraction
LVDd: Left ventricular end-diastolic diameter
LVDs: Left ventricular end-systolic diameter
LAD: Left atrial dimension
SpO_2_: Peripheral oxygen saturation
MAPE: Mean absolute percentage error
RNN: Recurrent neural network
LSTM: Long short-term memory network
ReLU: Rectified linear activation.
MSE: Mean square error
